# Characterization of early and terminal complement proteins associated with polymorphonuclear leukocytes *in vitro* and *in vivo* after spinal cord injury

**DOI:** 10.1186/1742-2094-5-26

**Published:** 2008-06-25

**Authors:** Hal X Nguyen, Manuel D Galvan, Aileen J Anderson

**Affiliations:** 1 Physical Medicine & Rehabilitation, 1105 Gillespie Neuroscience Research Facility, University of California, Irvine, CA 92697-4292, USA; 2 Anatomy and Neurobiology, University of California, Irvine, CA 92697-4292, USA; 3 Sue and Bill Gross Stem Cell Research Center, University of California, Irvine, CA 92697-4292, USA; 4 Reeve-Irvine Research Center, University of California, Irvine, CA 92697-4292, USA

## Abstract

**Background:**

The complement system has been suggested to affect injury or disease of the central nervous system (CNS) by regulating numerous physiological events and pathways. The activation of complement following traumatic CNS injury can also result in the formation and deposition of C5b-9 membrane attack complex (C5b-9/MAC), causing cell lysis or sublytic effects on vital CNS cells. Although complement proteins derived from serum/blood-brain barrier breakdown can contribute to injury or disease, infiltrating immune cells may represent an important local source of complement after injury. As the first immune cells to infiltrate the CNS within hours post-injury, polymorphonuclear leukocytes (PMNs) may affect injury through mechanisms associated with complement-mediated events. However, the expression/association of both early and terminal complement proteins by PMNs has not been fully characterized in vitro, and has not observed previously in vivo after traumatic spinal cord injury (SCI).

**Method:**

We investigated the expression of complement mRNAs using rt-PCR and the presence of complement proteins associated with PMNs using immunofluroescence and quantitative flow cytometry.

**Results:**

Stimulated or unstimulated PMNs expressed mRNAs encoding for C1q, C3, and C4, but not C5, C6, C7 or C9 in culture. Complement protein C1q or C3 was also detected in less than 30% of cultured PMNs. In contrast, over 70% of PMNs that infiltrated the injured spinal cord were associated with C1q, C3, C7 and C5b-9/MAC 3 days post-SCI. The localization/association of C7 or C5b-9/MAC with infiltrating PMNs in the injured spinal cord suggests the incorporation or internalization of C7 or C5b-9/MAC bound cellular debris by infiltrating PMNs because C7 and C5b-9/MAC were mostly localized to granular vesicles within PMNs at the spinal cord epicenter region. Furthermore, PMN presence in the injured spinal cord was observed for many weeks post-SCI, suggesting that this infiltrating cell population could chronically affect complement-mediated events and SCI pathogenesis after trauma.

**Conclusion:**

Data presented here provide the first characterization of early and terminal complement proteins associated with PMNs in vitro and in vivo after SCI. Data also suggest a role for PMNs in the local internalization or deliverance of complement and complement activation in the post-SCI environment.

## Background

The complement cascade includes at least 30 soluble and cell membrane bound proteins and has an important role in the modulation of inflammation and defense against infection. However, numerous complement proteins have been suggested to affect central nervous system (CNS) injury or disease by promoting inflammation, phagoctyosis, and cell lysis [1,2]. Additionally, the activation of complement via the classical or the alternative pathway results in the formation and deposition of C5b-9 membrane attack complex (C5b-9/MAC), consisting of complement proteins C5, C6, C7, C8, and C9, which can promote cell lysis or exert sublytic effects on target cells [3,4]. Although several studies have demonstrated the deposition of C5b-9/MAC on neurons and oligodendrocytes after traumatic CNS injury [5-7], the contributing source of complement proteins and activation at the local environment of trauma has not been clearly defined.

Proteins of the complement system are thought to originate mostly from the liver; however, previous studies have reported the expression or production of some complement proteins by other cell types from the CNS [8,9] and the immune system [10,11]. Although complement derived from numerous cell types could contribute to injury or disease in the CNS, infiltrating immune cells may represent an important local source of complement after injury. PMNs are the first peripheral immune cells to respond to CNS injury, infiltrating within hours. As such, PMNs have been suggested to promote injury after CNS trauma [12,13] and have been shown to promote neurotoxicity through mechanisms associated with matrix metalloproteinases (MMPs), reactive oxygen species (ROS), and tumuor necrosis factor α (TNF-α) [14]. Although PMNs are not known to affect injury or neurotoxicity through complement-mediated events or activation, a few studies have shown the expression or production of C3 and factor B by cultured PMNs in vitro [15-17]. In addition, several studies have suggested that exposure of cultured PMNs to biological or pharmacological stimulation induces the release of several critical terminal proteins (C6 and C7) in PMN cultures [18,19]; however, the expression of mRNAs encoding for C6 or C7 by PMNs has not been confirmed. Nevertheless, it has been reported that in some cases terminal complement proteins are synthesized by non-resident cell types at sites of inflammation. For example, a previous study has suggested that although hepatocytes synthesize the majority of proteins of the complement system, most C7 proteins generated after liver transplantation are synthesized by other cell types [20], suggesting a local delivery of complement proteins and the contribution of complement-mediated events by a non-hepatocytic cellular source at the site of inflammation.

Based on the data from previous studies, the potential of PMNs to deliver complement proteins locally and affect complement-mediated events or C5b-9/MAC formation has been suspected but has not been previously characterized fully in vitro or investigated in vivo after CNS injury. A better understanding of the association of PMNs with complement proteins may have critical implications for the potential role of PMNs in complement-mediated events or activation after CNS injury. The data presented below provide for the first time, a more complete characterization of early and terminal complement proteins associated with PMNs in vitro and in vivo after traumatic spinal cord injury (SCI).

## Methods

### Isolation of PMNs from rat peritoneal cavity

As described previously [21], adult female rats (Sprague Dawley) received an intraperitoneal injection of sterile 12% sodium caseinate solution and were sacrificed by isoflurane inhalation 16 hours later. The peritoneal exudate containing leukocytes was collected and red blood cells were lysed with 0.85% ammonium chloride, resuspended in Hanks' balanced salt solution (HBSS), overlaid on Histopaque 1077 (Sigma), and then centrifuged at 400 G for 45 minutes at 4°C. PMNs were pelleted and separated from other leukocytes, which partitioned at the HBSS-Histopaque interface. PMNs collected were cultured in conditions described below and identified by immunofluorescence and flow cytometry.

### Hydrogen peroxide production in PMN culture

Hydrogen peroxide was measured by a one-step assay that uses 10-acetyl-3,7-dihydroxyphenoxazine (Amplex Red reagent, Molecular Probes) to detect hydrogen peroxide concentration in PMN (5.0 × 10^6^/ml HBSS) culture. In the presence of horseradish peroxidase (HRP), Amplex Red reagent reacts with hydrogen peroxide to form a fluorescent product, resorufin, readable at 560 nm. Briefly and according a previous study [22], a volume of 100 μl reaction mixture containing 50 μM Amplex Red reagent and 0.1 U/ml HRP in Krebs-Ringer phosphate was mixed with a volume of 20 μl of Krebs-Ringer containing 1.5 × 10^4 ^PMNs, incubated at 37°C for 30 minutes, and was read at 560 nm with a fluorescence microplate reader equipped for excitation in the range of 530–560 nm and emission detection at ~590 nm.

### Expression of complement mRNAs by cultured PMNs

Expression of C1q, C3, C4, C5, C6, C7, C9, CD59, Crry, and GADPH mRNAs in cultured PMNs was assayed by rt-PCR. Briefly, mRNAs were isolated from rat peritoneal PMNs (5 × 10^6^/ml) after 24 hours in culture with no stimulant or with 0.64 μM phorbol myristate acetate (PMA, Sigma), TNF-α (2000 U/ml, Serotec), IFN-γ (2000 U/ml, Serotec), or a combination of TNF-α and IFN-γ. mRNAs isolated from rat liver cells were used as positive controls for all complement proteins and inhibitors, while negative controls were absent of mRNAs. mRNAs from PMNs or liver cells were isolated according to the RNeasy Mini Kit (Qiagen), and rt-PCR screening conditions and reagents were according to the OneStep RT-PCR Kit (Qiagen). For each rt-PCR reaction, 0.25 μg of mRNAs was used in thermal cycler conditions of 30 minutes at 50°C; 15 minutes at 95°C; followed by 30-repeated cycles of 1 min at 95°C, 1 min at 55°C, 1 min at 72°C; and ended with 10 minutes at 72°C. Primers used for rt-PCR are listed in Table 1 and are according to previous studies [23-26].

**Table 1:** Sequences of primers used for rt-PCR

	Forward (5' to 3')	Reverse (5' to 3')	Reference or Accession Number
C1q	CGACTATGCCCAAAACACCT	GGAAAAGCAGAAAGCCAGTG	Fan et al. 2004
C3	GACCTGCGACTGCCCTACTCT	CTGATGAAGTGGTTGAAGACG	X52477
C4	AGACCCGCAACTTTCTGGTCC	TGAGCTCAGGGATCCAGAGTC	U42719
C5	GACCTGCAGCTCCTGCATCAGA	CCTTCCCAACAGCATGCCTTTGT	X91892
C6	GGGGCAAGTATGACCTTCTC	TGGGGACCGTTTTTCACAGT	Qian et al. 1999
C7	CAGAATTCTGTCCATCACCTC	GAATTCTGCAG1TFCTCCCAG	Oka et al. 2001
C9	TAATGGTGACAACGACTGTGG	TGACAAGTAGGATGACAATCG	U52948
CD59	ATGCTACAACTGTTTAGACCC	CTTCGAAGCTTTTGTTACACAAGT	U48225
Crry	GTTCTTGATGGAGTGGAAAGC	CTGAGGCTGAACACAGCTGTC	Sohn et al. 2000
GADPH	TGAAGGTCGGTGTCAACGGATTTGGC	CATGTAGGCCATGAGGTCCACCAC	Sohn et al. 2000

### Traumatic injury to rat spinal cord

All rats (Sprague Dawley, adult female, 200 grams) were anesthetized with ketamine/xylazine (100/10 mg/kg i.p.) before receiving a moderate contusion injury (200 kilodynes) at T9, using an Infinite Horizons Impactor at the UC Irvine SCI Core Facility. All surgeries were done by personnel previously trained to perform consistent laminectomies and injuries. Gel foam was placed over the exposed spinal cord before suturing the muscle and closing the skin with wound clips. Post-operative animal care consisted of buprenorphine (50 mg/kg s.c.) twice daily for 2 days, lactated ringers (50 ml/kg s.c.) once daily for 5 days, Baytril (2.5 mg/kg, s.c.) once daily for 14 days, and manual bladder expression twice daily until animals were sacrificed at various time points within 42 days post-SCI. Spinal cord injured rats (n = 5 animals per group) were anesthetized with a lethal dose of sodium pentobarbital (0.4 ml) 30 minutes, 2 hours, 24 hours, 3 days, 7 days or 42 days-post SCI, while uninjured control rats received no injury to the spinal cord. Cord segments (T8-T10) containing the injury epicenter at T9 were then removed from all animals for immunolabeling or flow cytometric analysis. All work was done with the approval of the Institutional Animal Care and Use Committee at the University of California, Irvine.

### Immunolabeling

Cultured peritoneal PMNs (10^6^/ml) were adhered to microscope slides by centrifugation (Cytospin, Shandon, USA), and then fixed with acetone for 10 minutes before immunolabeled for PMN, C1q, C3, C7, or C5b-9/MAC. Briefly, cells adhered on slides were quenched in 0.3% hydrogen peroxide solution for 15 minutes and then incubated in blocking buffer (3% BSA and 0.05% Tween-20) for 30 minutes. Following several washes with PBS, cells were incubated in rabbit anti-rat PMN-FITC (1:500, Accurate Chemical) and goat anti-human C1q (1:500, Advanced Research Technologies (ART)), C3 (1:500, ART), murine anti-human C7 (1:500, Quidel) or C5b-9/MAC (1:500, Quidel) solution for 2 hours at room temperature. Immunoreactive complement proteins were identified using a donkey anti-goat or goat anti-mouse IgG conjugated with Alexa Fluor 555 (1:1000, Invitrogen). All cells were also identified using a nucleic-fluorescent dye, Hoechst 33342 (1:1000, Invitrogen). Antibody control slides were processed identically, except that incubation with primary antibody was eliminated.

Spinal cord segments containing the injury epicenter were dissected by T8-T10 spinal roots, sunk in 20% sucrose and 4% paraformaldehyde overnight at 4°C, frozen in isopentane at -56°C and then stored at -80°C. Cross sections (30 μm) of spinal cord tissue were cut on a sliding microtone. Serial sections were collected in PBS with sodium azide (0.02%) and stored at 4°C until use. Immunohistochemistry was performed as previously described with minor modifications [27]. Briefly, sections are washed with Tris (100 mM Tris-HCL, pH 7.4) and immediately exposed to 3% hydrogen peroxide and 10% methanol to inactivate endogenous peroxidases. The sections were then washed with Tris, then TBS-A (Tris pH 7.4 with 0.4% Triton ×) and blocked with TBS-B* (Tris + 2% BSA + 2% Donkey serum). Subsequently, the tissue sections were incubated with mouse anti-rat CD43 (1:1000, Serotec) in TBS-B* for 2 hours at room temperature, while antibody control sections were incubated in the absence of primary antibody. Sections were then washed with TBS-A to remove unbound antibody. Following the washes, the tissue sections were blocked with TBS-B* and then incubated in the appropriate biotinylated donkey secondary antibody (1:500, Jackson) in TBS-B* for 1 hour at room temperature. Sections were then washed with TBS-A to remove any unbound secondary antibody and then incubated in ABC reagent (Vector laboratories methodology) for 1 hour at room temperature. Following ABC exposure, the tissue sections were washed first with TBS-A, followed by two washes with Tris and then exposed to diaminobenzidine (DAB; Vector Laboratories) solution for 4 minutes to visualize formation of brown precipitate.

Tissue sections used for confocal imaging were prepared as described above and were incubated in rabbit anti-rat PMN-FITC (1:500, Accurate Chemical) and murine anti-human C7 (1:500, Quidel) or C5b-9/MAC (1:500, Quidel) in TBS-B* for 2 hours at room temperature. Following several washes, immunoreactive complement proteins were identified using goat anti-mouse IgG conjugated with Alexa Fluor 555 (1:1000, Invitrogen) in TBS-B* for 1 hour at room temperature. All cells were also identified using a nucleic-fluorescent dye, Hoechst 33342 (1:1000, Invitrogen). Confocal imaging was conducted using a Bio-Rad Radiance 2000 system and LASERSHARP 2000 software with lambda strobing to reduce bleed-through associated with simultaneous scanning.

### Flow cytometry

Spinal cord tissues (T8-T10) from five animals per group were dissociated in trypsin (0.5 mg/ml) and collagenase (1 mg/ml), and debris was separated from cells by centrifugation of cell suspension overlayed on OptiPrep gradient (Fisher) as described in previous studies [28,29]. Collected spinal cord cells or PMNs isolated from rat peritoneal cavity were incubated 5 minutes in 0.1% Triton ×-100 solution; 30 minutes in 100% normal rabbit, goat, or murine serum; 1 hour with rabbit anti-rat PMN-FITC (1:500; Accurate Chemical) and goat anti-human C1q (1:500, Advanced Research Technologies (ART)), C3 (1:500, ART), murine anti-human C7 (1:500, Quidel) or C5b-9/MAC (1:500, Quidel) diluted in HBSS; 1 hour with chicken anti-goat or rabbit anti-mouse Cy5 conjugated IgGs (1:500, Invitrogen) diluted in HBSS; and 30 minutes with propidium iodide (1:100; Molecular Probes) to label dead cells before analyzed on a FASC Calibur (Becton-Dickinson) flow cytometer. Cell viability was typically more than 90% and all flow cytometric gates were set using control IgG isotype labeled cells or spinal cord cells from uninjured animals. The mean values of cells positive for PMN, C1q, C3, C7, or C5b-9/MAC as determined by flow cytometry were expressed as percent (± s.e.) relative to control IgG isotype labeled cells or spinal cord cells from uninjured animals.

### Statistical analysis

All experimental groups were compared with their respective controls using unpaired Student's *t *tests. Statistical significance was defined at P < 0.05.

## Results

### Cultured PMNs express mRNAs of early complement but not terminal complement associated with C5b-9/MAC formation *in vitro*

Although previous studies have shown that cultured PMNs express mRNAs encoding for C3 and Factor B, it is not clear from these studies whether PMNs express other early complement proteins or terminal proteins necessary for C5b-9/MAC formation. The following experiments investigate whether cultured PMNs or PMNs stimulated with PMA, TNF-α, or IFN-γ express a complete spectrum of complement mRNAs, which may have important implications for a potential role of PMNs in complement-mediated events or the formation of C5b-9/MAC after SCI.

**Figure 1:**
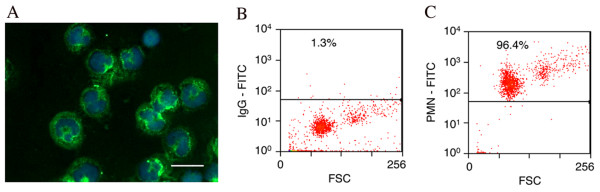
**Rat peritoneal neutrophils (PMNs) were identified by immunofluorescence (A, labeled with Hoechst (blue) and an anti-PMN anti-body (green)) and flow cytometry (C)**. All flow cytometric gates were set using control IgG isotype labeled cells (B). Over 95% of cells isolated were PMNs; scale bar = 10 μm.

**Figure 2:**
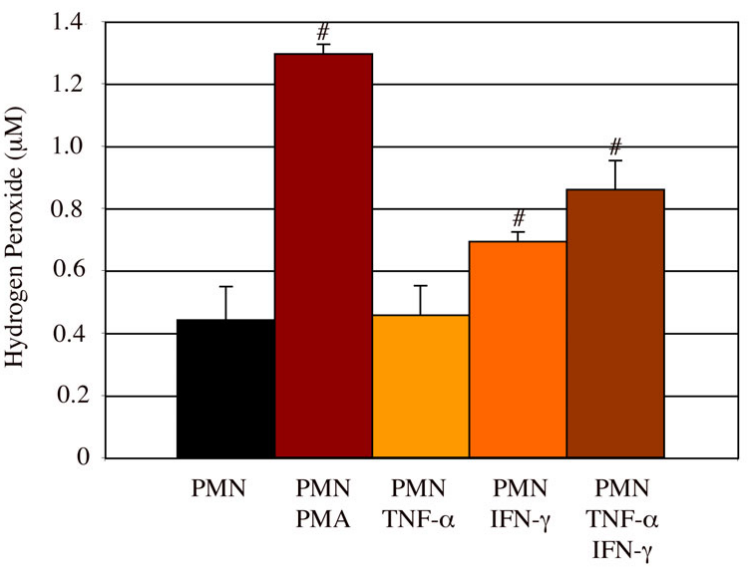
**Rat peritoneal PMNs were activated by PMA, TNF-α, or IFN-γ to release hydrogen peroxide**. PMNs (5.0 × 10^6^/ml) cultured with or without PMA (0.64 μM), TNF-α (2000 U/ml), or IFN-γ (2000 U/ml) stimulation released high levels of hydrogen peroxide. # = significantly different (P < 0.05) from PMN only. n = 6. Number of experiment replication = 3.

PMNs isolated from rat peritoneal cavity were confirmed to have 96% purity by immunofluorescence and flow cytometry (Figure 1). After 24 hours in culture with or without stimulation by PMA (0.64 μM), TNF-α (2000 U/ml), IFN-γ (2000 U/ml) or TNF-α + IFN-γ, PMNs were lysed and total RNAs collected for assay of mRNA expression of complement using rt-PCR. Activation of PMNs with stimulants was confirmed by the release of reactive oxygen species (hydrogen peroxide) in PMN cultures (Figure 2). PMA is a synthetic stimulant that diffuses into the cytoplasm of PMNs and activates protein kinase C (PKC) resulting in the subsequent generation of hydrogen peroxide. In contrast, cytokines such as TNF-α and IFN-γ bind specifically to receptors on PMNs, resulting in an increased uptake of calcium and the production of hydrogen peroxide by PMNs. The different mechanisms by which PMA and cytokines activate PMNs produced different levels of hydrogen peroxide in PMN culture, demonstrating that PMNs stimulated with PMA generated higher levels of hydrogen peroxide than those stimulated with TNF-α or IFN-γ by approximately two-fold (Figure 2).

**Figure 3:**
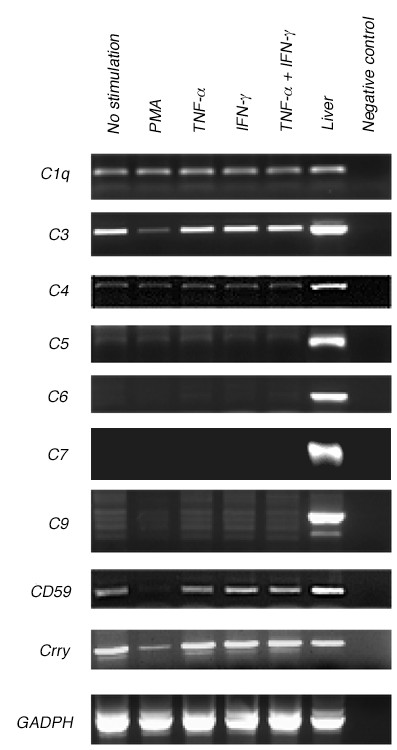
**Rat peritoneal PMNs express early proteins (C1q, C3, and C4) but not terminal proteins (C5, C6, C7, and C9) of the complement system, shown by rt-PCR**. mRNAs were collected from rat peritoneal PMNs (5 × 10^6^/ml) after 24 hours in culture with no stimulant or with PMA (0.64 μM), TNF-α (2000 U/ml), IFN-γ (2000 U/ml), or a combination of TNF-α and IFN-γ. Positive control mRNAs for all complement proteins and inhibitors (CD59 and Crry) of complement activation were isolated from rat liver, while negative controls were absent of mRNAs.

As shown in Figure 3 by rt-PCR, both unstimulated PMNs and stimulated PMNs expressed mRNAs encoding for C1q, C3, and C4, but not C6, C7, and C9 in culture. The faint C5 signal observed could have been due to either background noise or very low C5 expression by PMNs. In contrast, mRNAs of all complement proteins tested were detected in total RNA harvested from rat liver. As discussed above for hydrogen peroxide production, different activation mechanisms by PMA and cytokines may also account for the difference in C3 expression observed in this experiment, because in contrast to TNF-α or IFN-γ activation, PMA activation decreased C3 mRNA expression by PMNs (Figure 3).

Besides the early complement mRNAs, cultured PMNs also expressed mRNAs encoding for CD59 and Crry, which are inhibitors of complement-mediated lysis. As discussed above for C3, mRNA expression of these complement inhibitors by PMNs was also decreased by PMA activation, suggesting that PKC activity enhancement in response to PMA stimulation inhibits PMN expression of CD59 and Crry, and therefore, may contribute to the short lifespan of several days for PMNs. However, previous studies have shown that PMA and PKC activity enhanced CD59 expression in cancer cells and therefore, increased the survival of these cells [30,31]. The contrasting PMA-induced CD59 expression in PMNs and cancer cells suggests different responses by these cell types to PMA stimulation. Furthermore, PMN expression of mRNAs encoding for these complement inhibitors in several other states of activity affirms the detection of membrane-bound CD59 and Crry proteins on PMN surfaces as demonstrated in previous studies [32,33], supporting a self-protective role for these PMN-generated inhibitors of complement-mediated lysis under certain stimulating conditions. However, it is feasible that these PMN-generated inhibitors of complement-mediated lysis may also take part in the complex regulation of complement activation and function in the local injury environment.

### Cultured PMNs are associated with early complement proteins but not C5b-9/MAC *in vitro*

**Figure 4:**
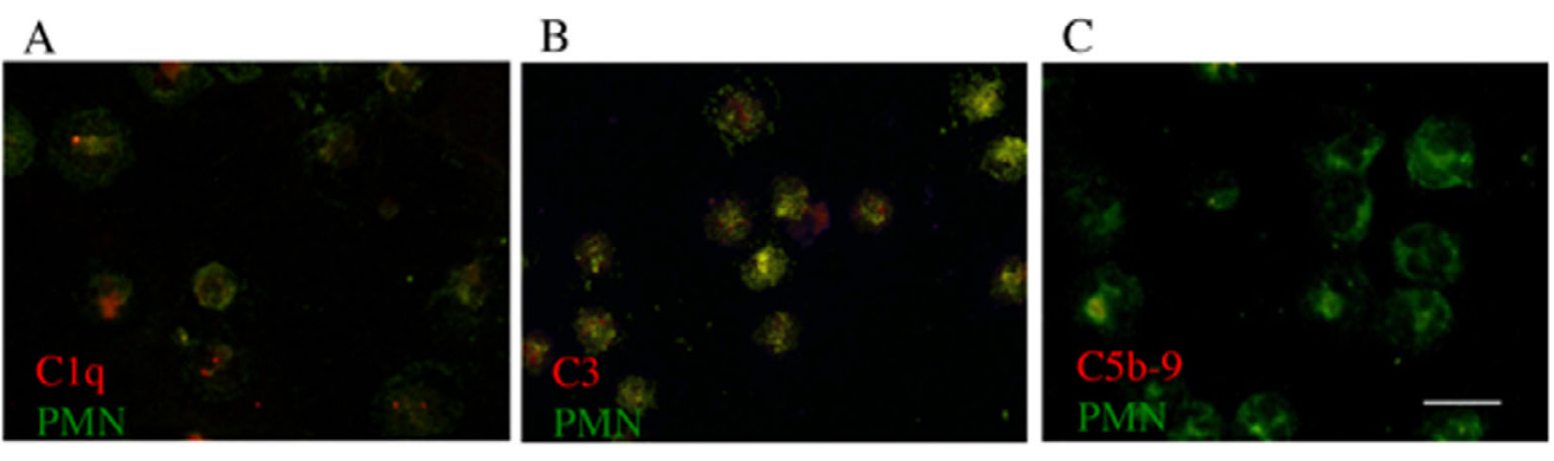
**Rat peritoneal PMNs are associated with the early proteins of the complement system but not C5b-9/MAC in vitro, shown by immunofluorescence**. PMNs were labeled with an anti-PMN anti-body (green) and an anti-C1q (A), anti-C3 (B), or anti-C5b-9/MAC (C) antibody (red). Scale bar = 10 μm.

**Figure 5:**
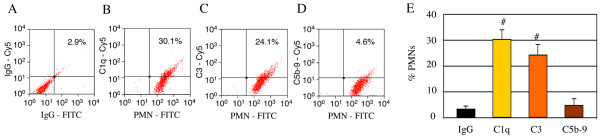
**Rat peritoneal PMNs are associated with the early proteins of the complement system but not C5b-9/MAC in vitro, shown by flow cytometry**. Cells (5 × 10^6^/ml) labeled with a FITC-conjugated anti-PMN antibody and a Cy5-conjugated antibody for C1q (B), C3 (C), or C5b-9 (D), or appropriate IgG controls (A) showed that nearly all cells were PMNs (B, C, & D), but only some cells were associated with C1q (B & E) and C3 (C & E); however, these cells were not associated with C5b-9/MAC (D & E). All flow cytometric gates were set using control IgG isotype labeled cells. # = significantly differ from IgG control. n = 3. Number of experiment replication = 3.

We have shown above that cultured PMNs express some complement mRNAs, we next ascertained whether these specific mRNAs are translated into proteins. Immunofluorescence of rat peritoneal PMNs using fluorescent antibodies for PMNs and C1q, C3, or C5b-9/MAC demonstrated that some, but not all cultured PMNs were associated with C1q and C3 proteins; however, as expected from the rt-PCR data above, cultured PMNs were negative for C5b-9/MAC immunofluorescent labeling (Figure 4). The association of complement proteins with cultured PMNs was also confirmed by flow cytometry (Figure 5). Cultured PMNs double-labeled with both a FITC-conjugated anti-PMN antibody and a Cy5-conjugated antibody for C1q, C3, or C5b-9/MAC, or appropriate IgG controls showed that nearly all cells were PMNs (as shown in Figure 1), and that 30.1% and 24.1% of cultured PMNs were associated with C1q (Figure 5B &5E) and C3 (Figure 5C &5E) respectively. In contrast, PMNs were negative for C5b-9/MAC (Figure 5D &5E), confirming the rt-PCR data above that showed a lack of mRNAs encoding for terminal complement proteins in cultured PMNs (Figure 3).

### PMN infiltration into the injured spinal cord peaks 24 hours and persists after acute traumatic SCI

Although complement-mediated events and lysis are generally thought to be part of a systemic response driven by the liver, there is increasing evidence suggesting that complement-mediated events/lysis are also driven locally by immune cells infiltrating sites of injury. As a principal immune cell to infiltrate the spinal cord after injury, PMNs may be an important source of complement. However, PMNs have not been shown previously to be associated with complement proteins in the injured spinal cord after SCI.

**Figure 6:**
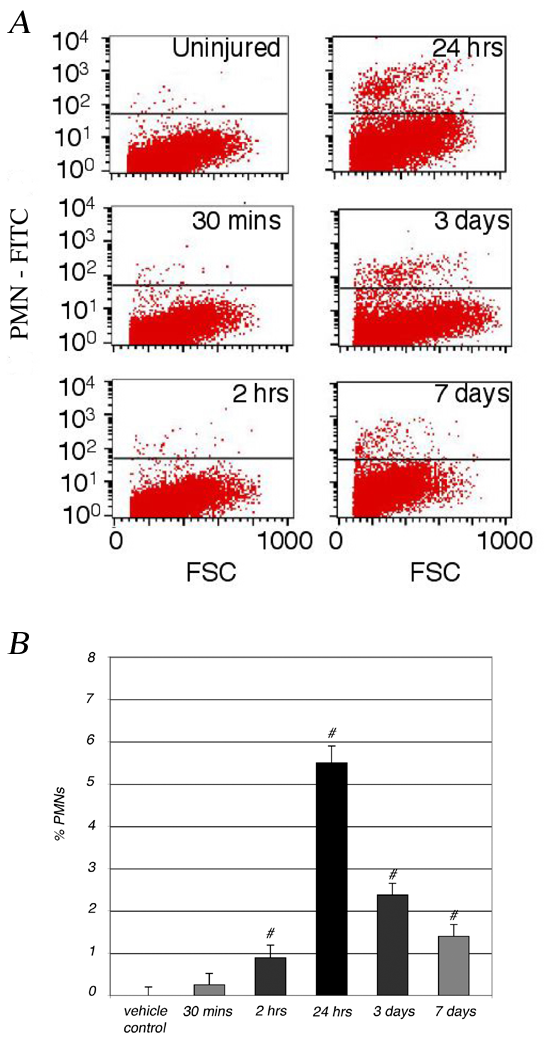
**Time course of PMN infiltration after a moderate SCI at T9, demonstrated by flow cytometry in the injured spinal cord**. Cells isolated from spinal cord (T8-T10) were labeled with a FITC-conjugated anti-PMN antibody before analyzed on a FACS Calibur flow cytometer. Spinal cord tissues were collected from uninjured control or injured animal 30 minutes, 2 hours, 24 hours, 3 days, or 7 days after SCI. The number of PMNs detected in the injured or uninjured spinal cord was visualized by dot plots (A), and represented as the percent of cells in the spinal cord relative to uninjured control (B). All flow cytometric gates were set using control IgG isotype labeled cells. # = significantly (P < 0.05) different from uninjured control. n = 5. Number of experiment replication = 2.

**Figure 7:**
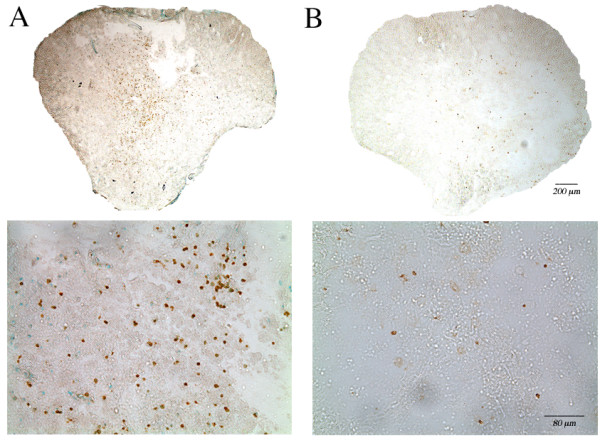
**PMN infiltration was observed at the spinal cord epicenter region after SCI**. Cross sections of rat spinal cord adjacent to the epicenter region were immunolabeled for PMNs 3 days (A) or 42 days (B) after a moderate contusion to the spinal cord (T9).

The time course of PMN infiltration in rat spinal cord tissue after SCI was investigated using flow cytometry to quantitate the number of PMNs in the spinal cord with respect to other CNS and infiltrating cells in the cord. Spinal cord epicenter tissues (segment T8-T10) collected from uninjured controls and spinal cord injured animals 2 hours, 24 hours, 3 days, or 7 days after SCI were dissociated and labeled with a FITC-conjugated PMN antibody, before cells were analyzed on a FACS Calibur flow cytometer (Figure 6A). With respect to uninjured control, PMNs were detected in the injured spinal cord beginning 2 hours (0.9% ± 0.3) post-injury and peaked 24 hours post-injury, at which time 5.5% ± 0.4 were PMNs (Figure 6B). At 3 days and 7 days post-SCI, PMN number decreased to 2.4% ± 0.3 and 1.4% ± 0.3 of total cells in the cord (T8-T10) respectively. Although previous studies have suggested that PMN infiltration is an acute event after SCI, PMN number could be detected in the spinal cord 6 months post-SCI, at approximately 3% of total cells as determined by flow cytometry (data not shown). Similarly and as shown by immunohistochemistry, there are elevated numbers of PMNs at the injury epicenter region of the cord 3 days post-SCI (Figure 7A) and smaller numbers of PMNs remain detectable 42 days post-SCI (Figure 7B), as quantified by flow cytometry and stereological quantitative assessment (data not shown). Together with in vitro data presented above showing the association of PMNs with some early complement proteins, the elevated PMN numbers at the injury epicenter region suggest that PMNs could contribute to increased complement-mediated events/lysis at the local injury environment.

### Infiltrating PMNs are associated with early complement proteins *in vivo *after traumatic SCI

**Figure 8:**
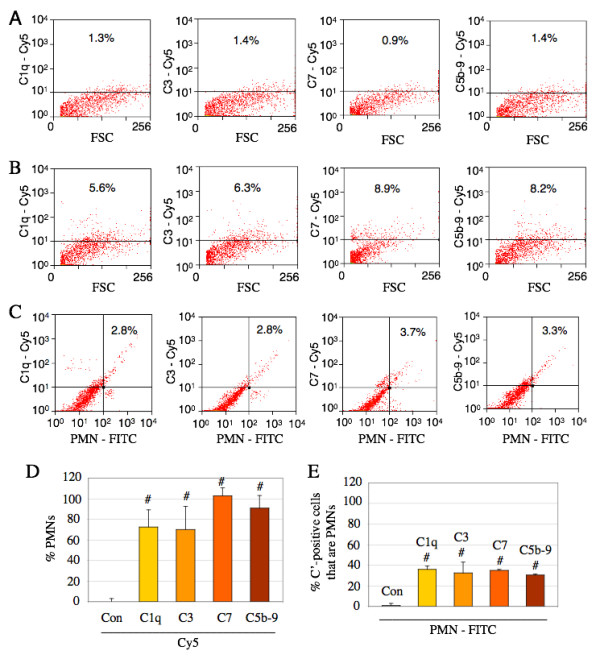
**Infiltrating PMNs in the injured spinal cord are associated with the early and terminal complement proteins 3 days post-SCI**. Cells isolated from spinal cord (T8-T10) of control (A) or injured rats (B & C) were labeled with a FITC-conjugated anti-PMN antibody and a Cy5-conjugated antibody for C1q, C3, C7 or C5b-9, or appropriate IgG controls before analyzed on a FACS Calibur flow cytometer. After 3 days following SCI (B), the number of cells positive for C1q, C3, C7, or C5b-9 was elevated compared to that from control (A). However, only some of the complement-positive cells were PMNs (C & E) and that most PMNs that infiltrated the injured spinal cord were also positive for C1q, C3, C7, and C5b-9 (C & D). All flow cytometric gates were set using control IgG isotype labeled cells. # = significantly (P < 0.05) different from uninjured control (Con). n = 5. Number of experiment replication = 2.

Although the data presented above show that cultured PMNs were associated with the early complement proteins in vitro, the association/co-localization of complement proteins with PMNs that have infiltrating into the injured spinal cord has not been previously characterized. The association of complement proteins with infiltrating PMNs was tested for the first time in vivo by flow cytometry, in which dissociated cells from injured spinal cord tissues were analyzed for PMN and complement markers (Figure 8). Most complement proteins detected immediately within 1 day post-SCI could likely originate from the serum as a result of the blood-spinal-barrier disruption; therefore, spinal cord tissues of rats 3 days post-SCI were used accordingly. Cells dissociated from spinal cord tissue (T8-T10) of uninjured controls (Figure 8A) or spinal cord injured rats (Figure 8B &8C) were labeled with a FITC-conjugated anti-PMN antibody and a Cy5-conjugated antibody for C1q, C3, C7 or C5b-9/MAC, or appropriate IgG controls before analyzed on a flow cytometer.

Three days following SCI (Figure 8B), the number of cells at the injury epicenter region (T8-T10) that were positive for early complement proteins (C1q or C3) in the spinal cord increased to 5.6% and 6.3% respectively, compared to 1.3% and 1.4% from uninjured controls (Figure 8A). However, more than 30% of total cells positive for C1q or C3 in the spinal cord epicenter were identified as PMNs (Figure 8C &8E). Therefore, while other cells besides PMNs were associated with C1q or C3 in the cord 3 days post-SCI, the infiltrating PMNs represented a significant fraction of this labeling. Additionally, of all the PMNs that infiltrating the spinal cord epicenter region, most (over 70%) were also positive for C1q or C3 (Figure 8C &8D). In contrast with the in vitro data above that showed less than 30% of cultured PMNs were associated with C1q or C3 proteins (Figure 5), data presented here show that after 3 days following SCI, 78% or 70% of infiltrating PMNs exhibited C1q or C3 respectively, suggesting an elevated number of infiltrating PMNs synthesizing or internalizing complement proteins in the injured spinal cord. These in vivo data support the in vitro data that show the association of cultured PMNs with most early complement proteins, providing important implications for a PMN role in the local deliverance of complement, contributing to the elevated complement levels and complement-mediated events in the injured spinal cord.

### Infiltrating PMNs are associated with the terminal complement proteins *in vivo *after traumatic SCI

In contrast to the data above that showed a lack of terminal complement expression by cultured peritoneal PMNs, the characterization of the injured spinal cord region of rats 3 days post-SCI show that most infiltrating PMNs were associated with labeling for C7 and C5b-9/MAC. As shown in Figure 8B, the number of cells positive for C7 or C5b-9/MAC in the spinal cord epicenter region increased to 8.9% or 8.2% respectively (Figure 8B), while 30% of total cells positive for C7 or C5b-9/MAC in the spinal cord were PMNs (Figure 8C &8E). A different analysis of the data (shown in Figure 8D) also clarified that although most infiltrating PMNs (more than 95%) were associated with C7 or C5b-9/MAC (Figure 8C &8D), other cell types were also positively labeled for these terminal complement proteins.

**Figure 9:**
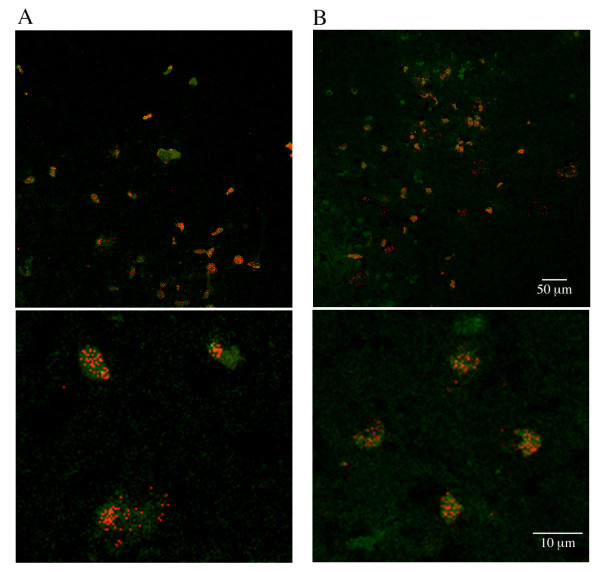
**PMNs are associated with C7 (A) and C5b-9 (B) at the injury epicenter region 3 days post-SCI**. Cross sections of rat spinal cord epicenter region were immunolabeled for PMNs (green) and C7 (red) or C5b-9 (red) 3 days after a moderate contusion injury to the spinal cord (T9). Lower panels are higher magnifications of selected areas taken from upper panels.

The association of C7 or C5b-9/MAC with infiltrating PMNs at the injury epicenter region was also confirmed by immunofluorescent confocal imaging of spinal cord cross sections 3 days post-SCI (Figure 9), demonstrating the co-localization of terminal complement proteins with PMNs. In most PMNs, terminal complement C7 (Figure 9A) and C5b-9/MAC (Figure 9B) appears to be localized in granular vesicles, suggesting that most PMNs may have incorporated/internalized these terminal complement proteins from the C5b-9/MAC bound cellular debris because, as shown in Figure 3, cultured PMNs lack the expression of terminal complement mRNAs.

In all, quantitative data of infiltrating PMNs associating with C1q and C3 after SCI support the demonstrated expression of early complement mRNAs and proteins by cultured peritoneal PMNs. However, although cultured peritoneal PMNs lack mRNA expression for terminal complement proteins, infiltrating PMNs were associated with terminal complement proteins in vivo 3 days post-SCI, suggesting the internalization of ingested complement by infiltrating PMNs after injury.

## Discussion

Data presented here provide the first characterization of early and terminal complement proteins associated with PMNs in vitro and in vivo after traumatic SCI. We have shown that cultured PMNs expressed mRNAs encoding for the early proteins C1q, C3, and C4, but not the terminal proteins C5, C6, C7, and C9 in vitro. Complement association with cultured PMNs was also demonstrated by immunofluroescence and flow cytometry, confirming that PMNs were associated with the early complement proteins that included C1q and C3. Additionally, we have characterized for the first time the localization/association of complement with the infiltrating PMNs in the injured spinal cord, demonstrating that the percentage of infiltrating PMNs that was associated with C1q or C3 in the injured spinal cord is twice as much as that detected in culture. More importantly and unlike PMNs in culture, most of the infiltrating PMNs were also associated with C7 and C5b-9/MAC in the injured spinal cord, suggesting that PMNs were incorporating/internalizing terminal complement proteins and C5b-9/MAC bound debris/damage cells at the injury microenvironment.

Although our assays did not detect mRNAs encoding for terminal complement proteins by cultured PMNs in our culture conditions, previous studies have suggested the storage and release of several terminal proteins by cultured PMNs in other culture conditions. These studies suggested that cell lysates and supernatant collected from cultured PMNs that were exposed to cytokines or C. albicans hyphae opsonized with normal human serum had elevated levels of C6 and C7 proteins [18,19]; however, it was not conclusive that these proteins were produced by PMNs in culture because (1) the rise in C6 or C7 in culture could have been released by C. albicans hyphae or derived from normal human serum, (2) the inhibition of protein biosynthesis by cycloheximide did not affect the rise in C6 or C7 in PMN cultures [18], and (3) conclusive evidence showing that PMNs are actively expressing these terminal complement mRNAs or proteins has been lacking in the literature. Our data show that cultured PMNs do not express mRNAs encoding for terminal complement proteins in vitro, even after exposure to numerous stimulants. The lack of terminal complement mRNAs in cultured PMNs may not have been unexpected because several studies have suggested that C6 and C7 are not actively synthesized by PMNs in culture, but were likely presynthesized in PMNs during differentiation in the bone marrow, stored in cytoplasmic vesicles, and subsequently released during circulation [18,34]. Although the concrete evidence of this occurrence has not been previously demonstrated, the likelihood of this occurrence can potentially clarify the lack of conclusive evidence showing active expression/generation of terminal complement mRNAs and proteins by cultured PMNs.

Contrary to our in vitro data showing the lack of terminal complement expression by cultured PMNs, our in vivo data show for the first time that infiltrating PMNs in the spinal cord 3 days post-SCI were associated with terminal complement proteins, suggesting that PMNs incorporated/internalized C5b-9/MAC bound debris. Our data also suggest the incorporation or internalization of C7 or C5b-9/MAC bound cellular debris because C7 and C5b-9/MAC associated with infiltrating PMNs were most likely localized to granular vesicles within PMNs at the spinal cord epicenter region. Furthermore, the localization of most C7 or C5b-9/MAC in the cytoplasm and not on the surface of PMNs suggests that these terminal complement proteins were not mediating lysis of PMNs. Moreover, data suggest that the association/localization of terminal proteins with infiltrating PMNs was likely attributed to the elevated internalization of C7 or C5b-9/MAC bound cellular debris in accordance with a phagocytic role, and has important implications for a PMN role in the local complement-mediated events at the injury epicenter region of the spinal cord. Alternatively, the proinflammatory microenvironment of the injured spinal cord could have induced a gain of phenotypic expression not observed in vitro.

Unlike the controversial expression/production of terminal proteins by PMNs, expression/production of C3 by PMNs has been clearly demonstrated in culture [16,17]. Our data confirm C3 expression by PMNs in culture, and demonstrate the expression of C1q and C4 by PMNs, providing a more complete characterization of PMN expression of the early complement proteins in culture. Furthermore, we have shown in vivo that most infiltrating PMNs (over 70%) in the spinal cord epicenter region 3 days post-SCI were associated with the immunolabeling of C1q or C3, compared to less than 30% of cultured PMNs in vitro. Our data suggest an increase in complement association with the infiltrating PMNs after SCI. Our data also provide important implications for the role of PMN-generated/internalized complement in numerous complement-mediated events after CNS injury. C1q has been suggested to affect stem cell migration and fate [35,36], while C3a and C5a have been suggested to promote and reduce neurotoxicity [37-40]. Moreover, previous studies have shown that C1q and C3 have high affinity for myelin [41-43], and previous work in our laboratory has shown that C1q and factor B co-localize with axons [5] and degenerating myelin (unpublished data) in the injured spinal cord. Degenerating myelin has been thoroughly demonstrated to inhibit axon growth and regeneration; however, the effect of complement bound to myelin after CNS injury is not known and has not been previously investigated. Although it is likely that the binding of complement opsonizes myelin debris for degradation, it is feasible that complement bound to myelin affects myelin inhibition of axon growth and regeneration. Moreover, glial cells of the CNS are known to express receptors for C3a and C5a, and therefore are likely to take part in the complement-mediated events following CNS injury [37,44]. The binding of C3a or C5a to receptors on oligodendrocytes and astrocytes may affect demyelination and scar formation following CNS injury, because (1) activation of C5aR by C5a peptide fragments has shown to trigger apoptosis in cortical and hippocampal cultures [40]; and (2) activation of C3a and C5a receptors on astrocytes has been suggested to regulating scar formation and glial cell activation/chemotaxis [45,46].

As the first predominant immune cells to infiltrate the CNS after injury, PMNs may significantly elevate the level of complement proteins/activation in the injured spinal cord. Previous studies have shown that the expression or display of complement proteins is prominantly detected in the CNS 24 hours after TBI [47,48] and SCI [5,49]. Co-incidentally, time course data of PMN infiltration in previous studies suggested that PMN numbers in the injured spinal cord also peaked 24 hours post-injury [50,51]; however, the localization/association of complement with the infiltrating PMNs in the injured spinal cord has not been previously characterized. Data in the present study confirm the peak of PMN infiltration 24 hours post-SCI and characterize for the first time, the association of complement with the infiltrating PMNs in the injured spinal cord, suggesting that PMNs may be an important contributor to this complement surge shortly post-SCI. However, other cells in the CNS (eg. astrocytes and neurons) have been shown to express/generate numerous complement proteins and may also contribute to this early complement surge after SCI [8,52,53]. CNS cells expressing complement proteins after SCI are widely distributed along the spinal cord outside of the injury epicenter, and a previous study has detected a surge of complement proteins/activation in the injured spinal cord as far as 20 mm rostral to injury epicenter [5]. In contrast, PMNs infiltrating the rodent spinal cord after injury are generally localized proximal to the epicenter, and neither we nor others have observed PMNs at large distances distal to this region.

The expression of early complement proteins by PMNs suggests that PMN-derived complement proteins may affect inflammation and other suggested alternative roles for complement. Animals that are deficient in C3 or received neutralizing antibodies or receptors for C1q or C3 have been shown to have less inflammation after CNS injury/disease, suggesting an important role for complement in recruiting inflammatory cells to sites of injury [54-56]. As suggested in the present study, complement delivered/internalized by PMNs at the injury site may affect the recruitment of macrophages and lymphocytes that infiltrating the injured CNS a few days later. Macrophages and T-cells have been suggested to affect both injury and regeneration after SCI and TBI [57-59]; therefore, the mediation of inflammation by PMN-delivered/internalized complement may have critical outcome to CNS injury and recovery. An alternative role for PMN-derived complement proteins could also affect neurogenesis and migration of neural progenitor cells (NPCs) in the injured CNS. A previous study has shown that NPCs express receptors for complement anaphylatoxins C3a and C5a, and has suggested that complement promotes neurogenesis because C3 (-/-) deficient mice and mice lacking C3aR or mice treated with a C3aR antagonist had impaired ischemia-induced neurogenesis [60]. As demonstrated in previous studies that showed PMNs synthesis and release of C3 in culture [16,17], and in the present study that showed PMNs express high levels of C3 mRNAs and proteins in vitro and in the injured spinal cord, it is likely that PMNs may contribute to the elevated level of complement involved in post-injury neurogenesis. Furthermore, the complement anaphylatoxins C3a and C5a are potent chemoattractants that could guide NPCs toward the site of injury; thus, the expression of C3a and C5a receptors by NPCs suggests the feasibility that complement may also affect NPC migration in the injured CNS. Critically, PMNs are mostly concentrated at the lesion site after SCI, and are expected to generate a few complement and derived fragments that may guide and direct NPC migration toward the site of injury.

## Conclusion

Although proteins of the complement system that are involved in complement activation and numerous other functions are thought to originate mostly from the liver, our data provide novel insight into an important cellular source of complement at the local microenvironment post-SCI. More importantly, the early presence of PMNs after injury and the long-term infiltration of PMNs at the injury epicenter for many months after SCI suggest that PMNs are likely to affect complement function and activation in the local SCI microenvironment.

## Competing interests

The authors declare that they have no competing interests.

## Authors' contributions

HXN and AJA were involved in the conception, design, analysis and interpretation of data, and the drafting of the manuscript, HXN and MG were involved in the troubleshooting of technical methods and acquisition of data. All authors read and approved the final version of the manuscript.
